# Type 2 diabetes affects bone cells precursors and bone turnover

**DOI:** 10.1186/s12902-018-0283-x

**Published:** 2018-08-08

**Authors:** Francesca Sassi, Ilaria Buondonno, Chiara Luppi, Elena Spertino, Emanuela Stratta, Marco Di Stefano, Marco Ravazzoli, Gianluca Isaia, Marina Trento, Pietro Passera, Massimo Porta, Giovanni Carlo Isaia, Patrizia D’Amelio

**Affiliations:** 1 Department of Medical Science, Gerontology and Bone Metabolic Diseases, University of Torino, Corso Bramante 88/90, 10126 Torino, Italy; 2 Department of Medical Science, Internal Medicine, University of Torino, Torino, Italy; 3 Geriatric Division, University of Turin, San Luigi Gonzaga Hospital, Orbassano, Turin, Italy

**Keywords:** Diabetes, Osteoblast, Osteoclast, Sclerostin, Receptor activator of nuclear factor κB, Bone density

## Abstract

**Background:**

Here we study the effect of type 2 diabetes (T2DM) on bone cell precursors, turnover and cytokines involved in the control of bone cell formation and activity.

**Methods:**

We enrolled in the study 21 T2DM women and 21 non diabetic controls matched for age and body mass index (BMI). In each subject we measured bone cell precursors, Receptor Activator of Nuclear Factor κB (RANKL), Osteoprotegerin (OPG), Sclerostin (SCL) and Dickoppf-1 (DKK-1) as cytokines involved in the control of osteoblast and osteoclast formation and activity, bone density (BMD) and quality trough trabecular bone score (TBS) and bone turnover. T2DM patients and controls were compared for the analyzed variables by one way ANOVA for Gaussian ones and by Mann-Whitney or Kruskal-Wallis test for non-Gaussian variables.

**Results:**

RANKL was decreased and DKK-1 increased in T2DM. Accordingly, patients with T2DM have lower bone turnover compared to controls. BMD and TBS were not significantly different from healthy controls. Bone precursor cells were more immature in T2DM. However the number of osteoclast precursors was increased and that of osteoblasts decreased.

**Conclusions:**

Patients with T2DM have more immature bone cells precursors, with increased number of osteoclasts and decreased osteoblasts, confirming low bone turnover and reduced cytokines such as RANKL and DKK-1. BMD and TBS are not significantly altered in T2DM although, in contrast with other studies, this may be due to the match of patients and controls for BMI rather than age.

## Background

Type 2 diabetes mellitus (T2DM) increases the risk of fragility fractures [1], even though it is often associated with increased bone density [1, 2]. T2DM has been associated with poor bone quality [3] and this may lead to increased fracture risk. Nevertheless, how T2DM affects bone is still controversial. Several mechanisms may be involved, such as direct effects of insulin resistance and hyperglycemia on the bone and bone marrow microenvironment, advanced glycation end products of bone matrix proteins, abnormal cytokine production, and impaired neuromuscular/skeletal interactions [4, 5]. Obesity associated with T2DM may be a confounder due to its controversial effect on bone per se (see Dolan et al., 2017 for a comprehensive review) [6]. Several studies suggest that obesity protects against bone loss in diabetic patients [7–9]. Moreover, recent data suggest that obesity, regardless of the presence of T2DM, is associated with a favorable bone microarchitecture and greater bone strength at the distal radius and distal tibia [10]. Serum markers of bone formation such as osteocalcin (OCN) and amino-terminal propeptide of procollagen type 1 (P1NP) have been found decreased in T2DM patients [11–13], supporting the hypothesis that bone formation is lower than in controls. Also bone resorption has been found reduced in T2DM by some authors [11, 14], however this data has not been confirmed by others [15]. T2DM may affect bone metabolism influencing osteoblast (OB) and osteoclast (OC) formation and activity by altering the cytokines involved in these processes other than having direct toxic effect on bone cells. OB formation and activity are mainly induced by the activation of the Wnt pathway, two of the most studied inhibitors of this pathway being sclerostin (SCL) and Dickoppf-1 (DKK-1) [16]. Otherwise, osteoclast formation and activity are mainly regulated by the Receptor Activator of Nuclear Factor κB (RANKL), its receptor (RANK) and its decoy receptor Osteoprotegerin (OPG) [17].

In vitro*,* in animal models and in humans it has been demonstrated that hyperglycemia increases the level of SCL [18–20] and DKK-1 [21–23], and that these cytokines blunt osteoblast formation and activity. As regards the RANKL/RANK/OPG pathway, this has been studied mainly in relation to cardiovascular damage and vascular calcification in T2DM [24]. Nowadays there are no human data on the relation between the cytokines involved in the control of bone cells and bone cell precursors in patients affected by T2DM. In this paper we show the effect of T2DM on bone turnover, bone precursors cells and cytokines involved in bone turnover taking into account the confounding factor of obesity and age.

## Methods

### Study population

We performed a case-control study enrolling 42 subjects, 21 women affected by T2DM and 21 non diabetic controls. Patients and controls had been in spontaneous menopause for, at least, one year. T2DM patients were matched with controls for Body Mass Index (BMI) ± 2 SD and age ± 5 years. Screening for micro-and macrovascular complications of diabetes was done yearly. Retinopathy was investigated by 45° digital retinal photography and graded according to the American Academy of Ophthalmology Simplified Classification [25]. Nephropathy was screened for by measuring albumin excretion rate and serum creatinine. Neuropathy was assessed according to the San Antonio Consensus [26]. Large vessel disease was screened for by examining peripheral pulses and history of coronary or peripheral artery disease. None of the T2DM patients included were affected by renal or macro-vascular complications, 4 were affected by retinopathy (19%). Of these patients,1 was also affected by neuropathy, and another 5 only had neuropathy (23.8%). (Table 1 shows the clinical characteristics of patients and controls). Five patients (23.8%) were treated by insulin, 11 by metformin and five by DPP4 inhibitors.

**Table 1:** Characteristics of subjects

	Patients (21)	Controls(21)	*P* value
Age (yrs)	71 ± 6	70 ± 6	–
Post-menopausalperiod (yrs)	22 ± 9	21 ± 7	NS
DMduration (yrs)	16 ± 2	–	–
HbA1C (mmol/mol)	57 ± 8.1	–	–
DM complications (%)	42.9%	–	–
Retinopathy (%)	14.3%	–	–
Neuropathy + retinopathy (%)	4.8%	–	–
Neuropathy (%)	23.8%	–	–
Insulin treatment (%)	23.8%		
Metformin treatment (%)	52.4%		
DPP4 inhibitors treatment (%)	23.8%		
Waist/hip ratio	0.92 (0.88–0.96)	0.88 (0.84–0.94)	NS
Fat mass (%)	39.4 (36.1–41.1)	39.1 (34.1–42.3)	NS
BMI (Kg/m^2^)	29 ± 5	29 ± 5	–

Data depicted are mean ± SD for Gaussian variables and median with 25° and 75° percentiles for non-Gaussian variables, non-continuous variables are shown percentage. Statistical differences were analyzed by using ANOVA one-way or Mann-Whitney U test

T2DM patients were recruited from the outpatient diabetes clinic of Medicina Interna 1 U. In Italy diabetic patients are managed by general practitioners and periodically referred to specialist centers to evaluate their disease state, hence the enrollment of patients from a tertiary referral center did not bias our results. Inclusion criteria for patients were:female genderin post-menopausal period and diagnosis of T2DM.

Exclusion criteria were: mental inability to sign the informed consent; known secondary osteoporosis; treatment with drugs active on bone turnover within the previous six months including: biphosphonates, strontium ranelate, parathyroid hormone, thyroid hormones, raloxifene, denosumab, corticosteroids, estrogen, oral anticoagulants, calcium and vitamin D andimmunosuppressant (as cyclosporine, azathioprine) within the previous year; diagnosis of type 1 diabetes; use of thiazolidinediones; history of cancer; liver disease, kidney failure (stage II or higher); malabsorption; hyperthyroidism.

Glycemic control in patients was measured by Hemoglobin A1C (HbA1C) with high performance liquid chromatography (HPLC), the mean level was 57 ± 8.1 mmol/mol.

Controls were recruited from the general population starting from the database used for our previous study, fully described elsewhere [27]. Briefly, controls were enrolled from the general practitioner lists amongst non-diabetic women without diseases active on bone metabolism, matched for age and BMI to T2DM patients, as previously described. Exclusion criteria were the same used for the patients. The whole population was Caucasian.

### Clinical evaluation of bone health

An accurate medical history, including the presence of fragility fractures, and physical examination was collected in all women. A bone scan was performed with a Hologic QDR 4500 X-ray densitometer to measure bone mineral density (BMD), both at lumbar spine and femur, and to evaluate the presence of vertebral fractures by morphometric DXA analyses. The spinal deformity index (SDI) [28] was calculated on DXA morphometry. Bone texture was analyzed by trabecular bone score (TBS) at lumbar vertebrae from DXA images with a dedicated software (TBS iNsight, Medimaps Group SA, Pessac, France). TBS is a textural index that evaluates pixel gray-level variations in the lumbar spine DXA image, providing an indirect index of trabecular microarchitecture. TBS is not a direct physical measurement of bone microarchitecture, but rather an overall score computed by the projection of the 3D structure onto a 2D plane that provides an indirect estimation of bone microarchitecture from spine DXA images [29].

### Bone turnover markers, cytokines and bone cells precursors

Markers of bone formation, OCN (Life Technologies Corp, Frederick, MD), P1NP (USCN, Life Science Inc. Houston, TX), and of bone resorption serum Tartrate Resistant Acid Phosphatase 5b (TRAP5b, Quidel, San Diego, CA) were measured by ELISA.

RANKL (Biovendor Research and Diagnostic Products, BRNO, Czech Republic), OPG (R&D Systems Inc., Minneapolis, USA), SCL (R&D Systems Inc., Minneapolis, USA) and DKK-1 (R&D Systems Inc., Minneapolis, USA) were also measured by ELISA.

To evaluate the role of circulating OC and OB precursors in T2DM, we measured them in peripheral blood mononuclear cells (PBMCs) separated by Ficoll-Paque technique [30]. Briefly, OC precursors were evaluated by staining PBMCs with fluorescein (FITC, supplied by B&D) conjugated anti-vitronectin receptor (VNR), phycoerythrin (PE, supplied by B&D) conjugated anti-CD14 and allophycocyanin (APC, supplied by B&D) conjugated anti-CD11b mAb, or with the corresponding isotype control, followed by incubation at 4 °C for 30 min as previously described [30]. Triple-positive cells (CD14+/CD11b+/VNR+) were regarded as osteoclast precursors, according to the literature [30, 31]. OB precursors were evaluated by staining PBMCs with FITC conjugated anti-CD15 (in order to exclude granulocytes expressing alkaline phosphatase, supplied by e-Bioscience), APC conjugated anti-alkaline phosphatase (ALP, supplied by R&D System Inc), PE conjugated anti-OCN (supplied by R&D System Inc), or with the corresponding isotype control, followed by incubation at 4 °C for 30 min as previously described [30–32]. CD15-/ALP+/OCN+ cells were regarded as osteoblast precursors according to the literature [30–32]. Membrane antigen expression was analyzed with the CellQuest software (Becton Dickinson & Co).

### Fat mass

In order to compare patients and controls for body fat mass and distribution, body fat was assessed by plicometry (Mahr GMBH Esslingen). The Pollock, Schmidt and Jackson’s formula was used on three sites (triceps, subscapular and abdomen) to calculate fat percentage [33]. In order to calculate BMI the women were weighted with a precision scale and their height recorded with a wall-mounted altimeter. BMI was measured as weight in Kg/squared height in meters, to evaluate fat distribution the waist/hip ratio was measured.

### Statistical analyses

The sample size was calculated to provide an 80% power (*p* < 0.05) to detect a 2-fold difference in SCL and DKK-1 in T2DM compared to healthy controls. The 2-fold difference was chosen based on previous papers [18–23]. In order to correctly weight the other data obtained the sample calculated post-hoc to evaluate differences in BMD to provide an 80% power (*p* < 0.05) to detect a 0.140 g difference in BMD in T2DM compared to healthy controls48 patients per group will be necessary. The 0.140 g difference was chosen based on previous papers [1, 2]. The sample size needed to evaluate differences in TBS to provide an 80% power (*p* < 0.05) to detect a 0.05difference in TBS in T2DM compared to healthy controls 100 patients per group will be necessary. The 0.05 difference was chosen on the basis of a previous paper [34]. The sample size needed to evaluate differences in bone turnover and in particular in P1NP to provide an 80% power (*p* < 0.05) to detect a 8 ng/mL difference in T2DM compared to healthy controls 33 patients per group will be necessary. The 8 ng/mL difference was chosen on the basis of previous paper [35].

T2DM patients and controls were compared by one-way ANOVA for Gaussian variables, by Mann-Whitney or Kruskal-Wallis test for non-Gaussian variables. Gaussian distribution was evaluated by kurtosis test. Gaussian variables were correlated by Pearson’s coefficient, non-Gaussian with Spearman correlation. Data were tested for outliers with the ROUT method, no outliers were identify and removed from the analyses. Statistics were performed by means of SPSS 24.0 for windows, Graph Pad Prism 7.0 for windows was used to drawn the graphs. *P* values were considered significant if lower than 0.05.

## Results

### T2DM affects bone precursors cell

**Fig. 1:**
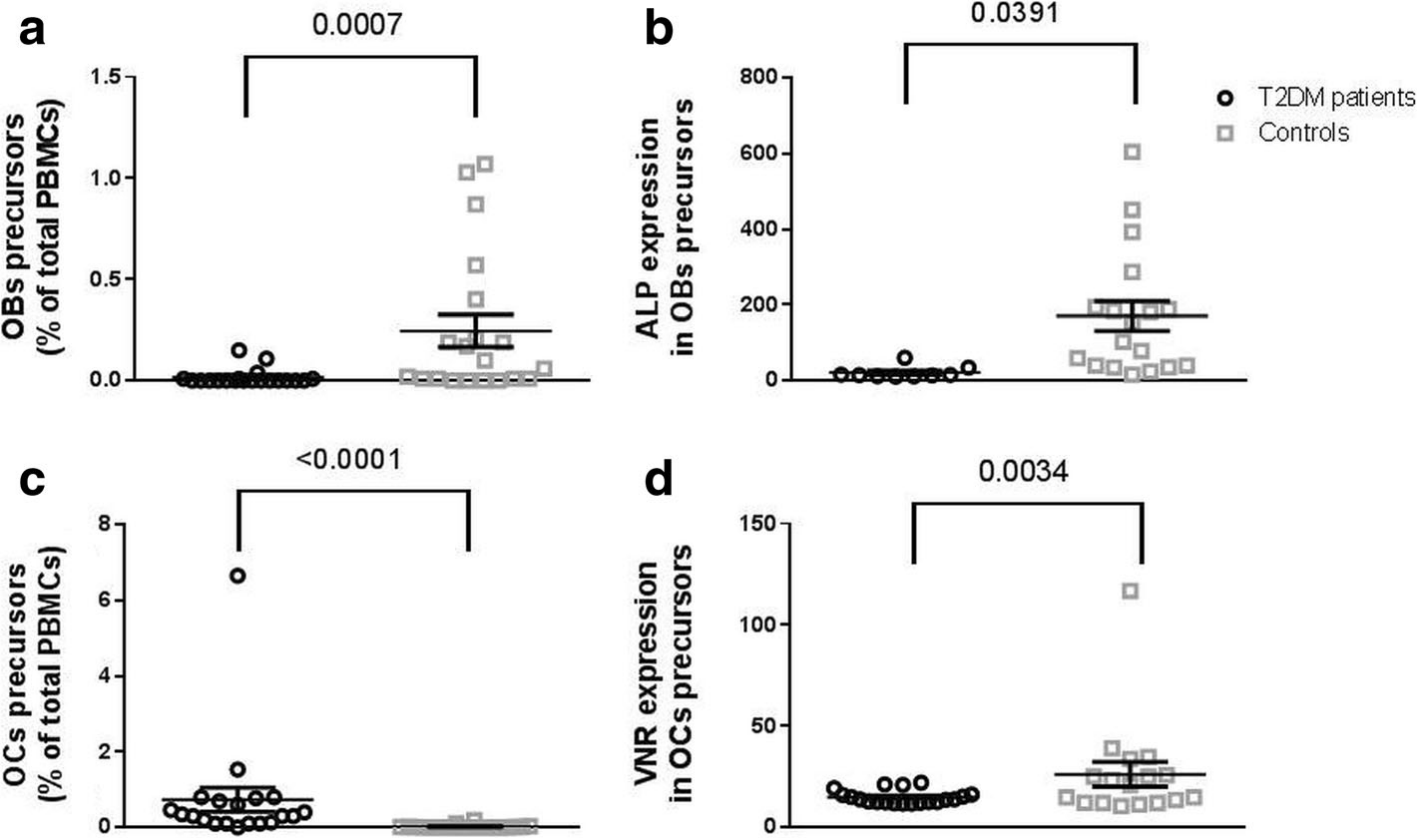
Dot plots show bone cell precursors in peripheral blood in T2DM patients and controls. Panel **a**: OB precursor cells; Panel **b**: ALP expression by OB precursor cells as measured by flow cytometry; Panel **c**: OC precursor cells; Panel **d**: VNR expression by OC precursor cells as measured by flow cytometry. *P* value was calculated with by one way ANOVA and is shown in the graph when significant

To evaluate if T2DM affects circulating bone precursors cells, we measured circulating OB and OC precursor cells and cytokines involved in osteoclastogenesis, osteoblastogenesis and in the regulation of bone turnover. We observed a significant reduction of circulating OB precursors cells in T2DM patients compared to controls (Fig. 1a), whereas OC precursors are increased (Fig. 1c). Both OC and OB precursors are more immature in diabetic patients; in particular OBs express lower levels of ALP and OCs express lower levels of VNR (Fig. 1b, d).

**Fig. 2:**
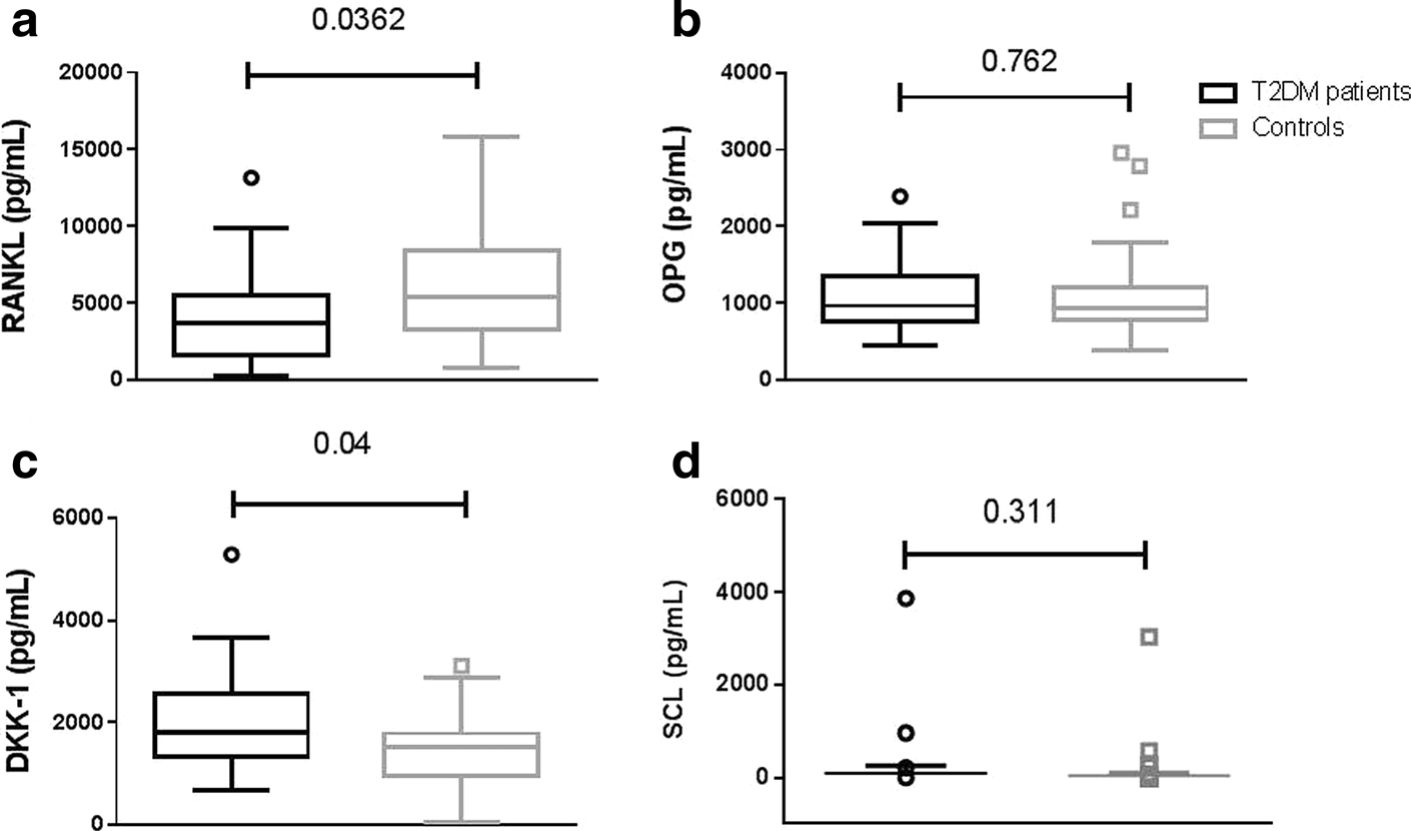
Graphs show cytokines involved in the control of bone cells formation and activity in T2DM patients and controls. Panel **a**: RANKL; Panel **b**: OPG; Panel **c**: DKK-1. Panel **d**: SCL. Box and whiskers plot displays median, the first and third quartiles, and the minimum and maximum of the data. P value was calculated with by Mann-Whitney test and is shown in the graph when significant

Cytokines involved in the regulation of bone cells are altered in T2DM patients: DKK-1 was increased in patients compared to controls (*p* = 0.04), whereas RANKL was decreased in T2DM (*p* = 0.0362). DKK-1 was 1824 pg/mL (1345–2572 interquartile range (IQR)) in T2DM versus 1526 pg/mL (963.2–1792 IQR) in the control group; RANKL was 3590 pg/mL (1434–7154 IQR) in T2DM versus 5018 pg/mL (2632–9343 IQR) in the control group (Fig. 2a, c). OPG was not significantly altered 965.2 pg/mL (759.1-1346IQR) in T2DM versus 938 pg/mL (783–1207 IQR) in the control group (Fig. 2b). SCL was undetectable in the majority of both patients’ and controls’ sera 561.3 ± 73.4 pg/mL in T2DM versus 309.8 ± 31 pg/mL (Fig. 2d). In three T2DM and 5 controls SCL was detectable in the serum, in those subjects bone formation measured by P1NP was significantly lower (12,420.6 ± 6706.1 vs 24,025.2 ± 992.9, *p* = 0.003), no other differences in the tested variables were detectable. The increased level of SCL may be related to decreased bone formation measured by P1NP.

Age per se is weakly correlated with RANKL (*R* = 0.32, *p* = 0.047) and with OB precursors maturation (*R* = − 0.384, *p* = 0.048). Post-menopausal state is directly correlated with RANKL (*R* = 0.323, *P* = 0.045). Other parameters are not influenced by age, post-menopausal state or by BMI.

Glycemic control measured by HbA1C did not correlate with bone cell precursor percentage and maturation, nor with cytokines involved in the control of bone turnover. There were no significant differences in the parameters analyzed in patients with or without diabetic complications and between patients taking different anti-hyperglycaemic drugs (data not shown).

### Bone metabolism is impaired in T2DM patients

BMD measured at lumbar spine, femoral neck and total femur was not significantly different between patients and controls; even though lumbar BMD was, on average, higher in T2DM than in controls. Bone structure measured by TBS, as well as SDI, were not altered in diabetic patients compared to controls (Table 2).

**Table 2:** Bone health in T2DM patients and controls

	T2DM patients (21)	Controls (21)	*P* value
Lumbar BMD (g/cm2)	0.97 ± 0.16	0.92 ± 0.15	0.059
FemoralBMD (g/cm2)	0.71 ± 0.12	0.69 ± 0.11	0.275
SDI	0 (0–1)	0 (0–1)	0.982
TBS	0.926 (0.799–1.027)	0.965 (0.766–1.051)	0.875

Data depicted are mean ± SD for Gaussian variables and median with 25° and 75° percentiles for non-Gaussian variables. Statistical differences are analyzed by using ANOVA one-way or Mann-Whitney U test

**Fig. 3:**
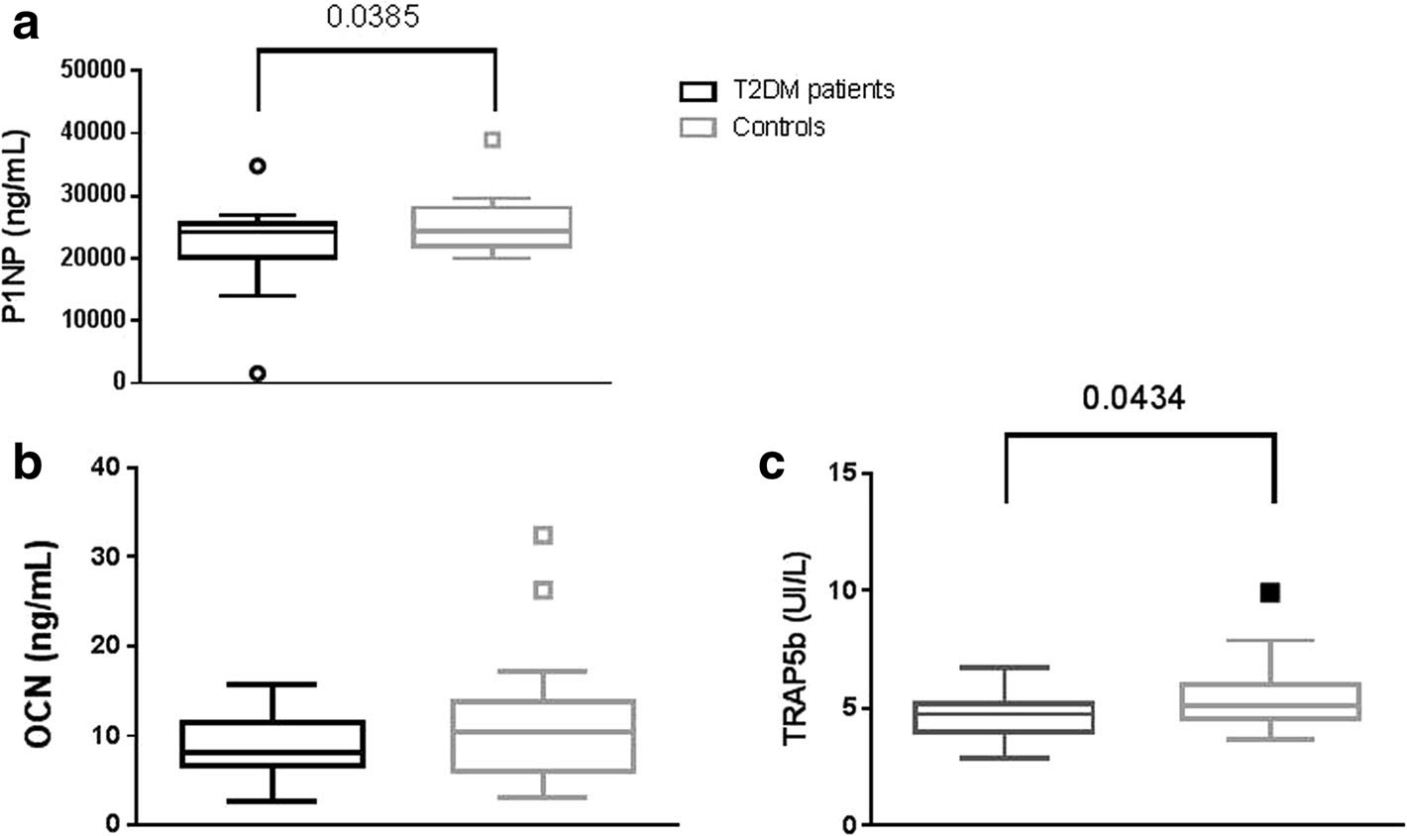
Graphs show bone turnover markers in T2DM patients and controls. Panel **a**: the bone formation marker P1NP; Panel **b**: the bone formation marker OCN; Panel **c**: the bone resorption marker TRAP5b. Box and whiskers plot displays median, the first and third quartiles, and the minimum and maximum of the data. P value was calculated with by Mann-Whitney test and is shown in the graph when significant

Obesity influences bone per se as there were significant correlations between BMI, BMD and TBS, the distribution of fat influenced only TBS (Table 3). Bone formation measured by P1NP as well as bone resorption measured by TRAP5b were significantly decreased in T2DM (Fig. 3). Glycemic control measured by HbA1C influenced bone structure but not bone density (Table 3). As regards bone turnover markers, HbA1C was inversely correlated with bone formation measured by OCN (*R* = − 0.59, *p* = 0.005).

**Table 3:** Correlations between bone density and structure, obesity and glycemic control

		BMI	Fat mass	Waist/hip	HbA1C
Lumbar BMD	r	**0.23**	0.84	0.91	−0.35
	p	**0.005**	0.338	0.276	0.286
Femoral BMD	r	**0.27**	0.154	0.10	−0.092
	p	**0.001**	0.078	0.904	0.701
TBS	r	**−0.319**	−0.36	**−0.34**	**− 0.55**
	p	**< 0.0001**	0.693	**< 0.0001**	**0.016**

Pearson’ coefficient correlations between BMD measured at lumbar spine and at femoral neck and BMI, Fat mass % and waist/hip ratio in the whole population under study, TBS was correlated by Spearman coefficient. Correlations between bone parameters and HbA1C were run only in T2DM patients. Significant values are in bold

## Discussion

The detrimental effect of T2DM on bone is well established [1, 2], but the possible mechanisms through which this happens have not been clearly elucidated. Here we evaluated the effect of T2DM on bone precursor cells and cytokines in patients and controls matched for BMI as well as age. One of the most confounding factor in the evaluation of diabetes effect on bone health is obesity, which is often associated with T2DM and has controversial effect on bone metabolism and fracture risk per se. Some studies suggest that obese subjects have a lower risk of proximal femur and vertebral fracture compared to adults with normal BMI [36, 37]. However the risk of fracture in obese subjects is variable at different skeletal sites according to the difference in falling mechanisms in these patients; in particular the risk for proximal humerus, upper leg and ankle fracture is higher in obese than in non-obese adults [38]. Moreover, increased fat mass could be detrimental to bone due to increased inflammation and production of adipokines that affect bone turnover [39, 40]. For these reasons, we enclosed in this study controls matched with patients for BMI as well as for age. The use of obese controls may explain why, differently from other studies, we did not find significant differences in bone microarchitecture measured by TBS between T2DM patients and controls. Although our study was not powered to measure differences in TBS [3, 41], our data show that obesity is inversely correlated with bone quality measured by TBS.

Here we show that osteoblast precursors cells are decreased and more immature according with decreased bone formation and increased DKK-1, whereas OC precursors are increased in the peripheral blood of T2DM patients. Data on OCs seem to be in contrast with decreased bone resorption in patients. However, it should be underlined that these are immature cells, which may not be able to home in bone microenvironment. Low RANKL levels in patients may explain the low grade of OCs maturation and decreased bone resorption.

This is the first study to evaluate bone cell precursors in the peripheral blood of diabetic patients.Previous data ina diabetic mouse model suggested reduced osteoclast and osteoblast formation in bone microenvironment [42]. An elegant in vitro study suggests that osteoclastogenesis mediated by RANKL is impaired in the presence of high glucose levels [43].

The increase in DKK-1, a well-known negative regulator of bone formation, may explain the decrease in bone formation in T2DM and confirms previous reports [18–20]. On the contrary, SCL was mostly undetectablein our cohort of patients. In the patients with detectable level we found a decreased bone formation without any other differences in the variables measured. Several studies investigated the levels of SCL in diabetic patients reporting conflicting results. Gennari et al. [44] showed increased levels of SCL in T2DM, but not in Type 1 diabetes mellitus (T1DM); other studies reported increased SCL in T2DM [45–47]. A recent study on post-menopausal women showed no difference between diabetic and non-diabetic patients in SCL levels [48]. In our study we evaluated only post-menopausal obese subjects, and this may be the reason why we achieved different results from other studies which included younger, leaner populations, also including men. Glycemic control, the use of different anti-hyperglycaemic drugs and the presence of diabetic complications did not appear to bias our results. Poor glycemic control may influence the levels and activity of cytokines active on bone turnover, some studies demonstrated that OPG is increased in T2DM and T1DM patients regardless to their glycemic control [49, 50], this finding is controversial as another study shows a reduction in OPG in T1DM patients [51], here we do not find any significant increase in OPG regardless to glycemic control. RANKL levels seem not to be influenced by glycemic control as shown by Lappin and colleagues [49], we found a decreased RANKL level without any correlation with glycemic control. SCL levels were not studied in relation with glycemic controls in previous studies [20, 44] here we do not find any relationship between glycemic control and SCL.

As regards clinical evaluation of bone health, we did not find a significant increase in BMD in T2DM compared to controls, in contrast to previous results [1, 2]. However, our cohort was small and the use of obese controls may have influenced this result as BMI per se, regardless of T2DM, is directly correlated with BMD both at lumbar spine and femoral neck.

As regards bone turnover, we found a significant decrease in bone formation and bone resorption in T2DM, confirming other studies [22, 35, 52].

The study was not powered to detect differences in fracture prevalence, hence the similar SDI between T2DM and controls may be due to chance. Age was weakly correlated with RANKL, as expected, and – interestingly – inversely correlated with OB precursor maturation. Use of controls matched with patients for age and BMI excludes this as a confounding factor.

Our study has several strengths and limitations. The analyses of bone turnover and related controlling cytokines was performed in well-characterized cohorts of patients and matched controls. This is the first study evaluating the role of bone cell precursors in T2DM. The significance of our findings may be limited by the small sample size and lack of measurement of parameters related to inflammation and adipocytokines production, some of the results reported may be flawed by the insufficient power.

## Conclusion

We show that bone precursor cells are affected by T2DM and, in particular there was a reduction of OB precursors and an increase in OC precursors. Both cell types appear to be more immature in T2DM, and this could be explained by increased levels of DKK-1 and decreased levels of RANKL.

## Abbreviations

ALP: Alkaline Phosphatase
APC: Allophycocyanin
BMD: Bone Mineral Dansity
BMI: Body Mass Index
DKK-1: Dickkopf-related Protein 1
FITC: Fluorescein Isothiocyanate
HbA1C: Hemoglobin A1C
HPLC: High Performance Liquid Chromatography
IQR: Interquartile Range
OB: Osteoblast
OC: Osteoclast
OCN: Osteocalcin
OPG: Osteoprotegerin
P1NP: Procollagen Type 1 Amino-terminal Propeptide
PBMCs: Peripheral Blood Mononuclear Cells
PE: Phycoerythrin
RANK: Receptor Activator of Nuclear Factor Kappa-Β
RANKL: Receptor Activator of Nuclear Factor Kappa-Β Ligand
SCL: Sclerostin
SDI: Spinal Deformity Index
T1DM: Type 1 diabetes mellitus
T2DM: Type 2 diabetes mellitus
TBS: Trabecular Bone Score
TRAP5b: Tartrate-resistant Acid Phosphatase 5b
VNR: Vitronectin
